# Transposition of the Mandibular Right Second Premolar Into the First Molar Space: A Case Report

**DOI:** 10.1155/crid/3829192

**Published:** 2026-11-04

**Authors:** Sura Ali Ahmed Fuoad Albayati, Mohammed Issam Al Hilwani, Faris Mohammad Abdoh

**Affiliations:** ^1^ Diagnostic and Surgical Dental Science Department College of Dentistry Gulf Medical University Ajman UAE gmu.ac.ae; ^2^ Gulf Medical University Ajman UAE gmu.ac.ae

**Keywords:** case report, mandibular premolar, panoramic radiography, rare dental anomaly, tooth transposition

## Abstract

**Background:**

Tooth transposition is an uncommon dental anomaly involving the positional interchange of two adjacent teeth within the same quadrant. Transpositions affecting posterior teeth in the mandible, particularly a premolar and a molar, are rarely reported, and their diagnosis and management can be challenging.

**Case Presentation:**

A 39‐year‐old man presented with difficulty in mastication related to multiple edentulous spaces in both arches. Clinical examination revealed poor oral hygiene, generalized gingival recession, Fordyce granules, a fissured tongue, and spacing of the maxillary anterior teeth. The mandibular right second premolar was found positioned within the first molar region, an incidental finding on intraoral examination. Periapical and panoramic radiographs confirmed the anomalous position of the premolar and demonstrated generalized alveolar bone loss. No reliable history could establish whether the first molar had previously been extracted, was congenitally absent, or had failed to erupt, and no family history of similar dental anomalies was reported. Management addressed the patient′s primary complaint through scaling and oral hygiene instruction, restoration of carious teeth, and prosthetic rehabilitation of edentulous spaces; no intervention was directed at the positional anomaly itself, as it was asymptomatic and of no functional or esthetic concern to the patient.

**Conclusion:**

This case illustrates a rare mandibular premolar–molar positional anomaly discovered incidentally in an adult, a presentation most consistent with tooth transposition. It highlights the value of thorough clinical and radiographic examination in identifying atypical dental findings and the importance of longitudinal or cross‐sectional imaging in confidently distinguishing true transposition from other causes of anomalous tooth position, such as postextraction migration.

## 1. Introduction

Tooth transposition, also termed dental translocation, is an uncommon anomaly in which two adjacent teeth within the same quadrant develop or erupt in each other′s positions [1, 2]. It represents a distinct form of ectopic eruption and may be complete, involving both crown and root, or incomplete, in which only the crown occupies the abnormal position while the root remains in its native location [2, 3].

Reported prevalence ranges from approximately 0.3%–1.4% of the general population, varying with the population studied and the diagnostic criteria applied [1, 4]. A female predilection and left‐side predominance have been described [1, 5]. Maxillary canine–first premolar transposition accounts for the large majority of reported cases; mandibular transpositions are far less common and almost always involve the canine and lateral incisor [1, 4]. Involvement of posterior teeth is exceptionally rare—in a meta‐analysis of 120 reported cases of tooth transposition, first–second molar involvement accounted for under 1% of cases, and transpositions specifically between a premolar and a molar are only anecdotally described in the literature [1].

The etiology of transposition is multifactorial, implicating genetic predisposition, interchange of adjacent tooth germs during odontogenesis, local trauma, and prolonged retention or early loss of the primary predecessor [2, 3]. When recognized early, interceptive orthodontic measures can be considered; in adults, in whom development is complete, management is instead guided by functional, esthetic, and periodontal considerations [2, 6].

This report describes an adult patient in whom the mandibular right second premolar was found positioned within the first molar region, an incidental finding during evaluation for an unrelated complaint. Because posterior mandibular transpositions of this type are so infrequently documented, we describe the clinical and radiographic findings and discuss the diagnostic considerations relevant to distinguishing true transposition from other causes of anomalous tooth position, such as postextraction migration.

## 2. Case Presentation

A 39‐year‐old Pakistani male presented to the Department of Oral Medicine at Thumbay Dental Hospital with a chief complaint of difficulty in mastication attributable to multiple edentulous areas in both arches. His medical history was noncontributory, with no reported systemic illness. He reported no family history of similar dental anomalies among first‐degree relatives.

The patient reported a 20‐year history of chewing Chalia, a form of smokeless tobacco, for approximately 30 minutes daily; he had discontinued this habit 3 years earlier but continued to place smokeless tobacco intraorally briefly, 5–6 times per day. He could not recall whether the mandibular right first molar had previously been extracted, had failed to erupt, or was congenitally absent, and no prior dental records were available to clarify this history.

Facial examination showed a mesocephalic cranial type and mesoprosopic facial form, with no facial asymmetry; the profile was straight and facial proportions were within normal anthropometric limits.

Intraoral examination revealed poor oral hygiene with heavy calculus deposits (particularly on the lingual surfaces of the mandibular anterior teeth), generalized gingival recession, Fordyce granules on the buccal mucosa, and spacing of the maxillary anterior teeth (Figure 1). The tongue showed irregular, shallow, asymptomatic fissuring (Figure 2A). Despite the patient′s tobacco use, no leukoplakic or erythroplakic changes, ulceration, or other mucosal pathology were observed.

**Figure 1:**
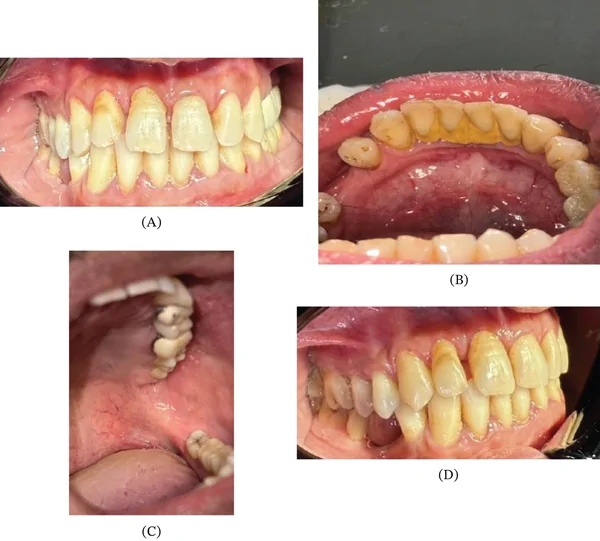
(A) Poor oral hygiene. (B) Calculus deposition. (A, D) Gingival recession and teeth spacing. (C) Fordyce granules on the buccal mucosa.

**Figure 2:**
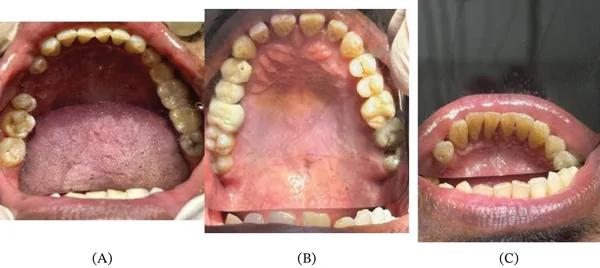
(A) Irregular tongue fissuring and translocation of the second mandibular premolar with the first molar. (B–C) Postscaling and oral prophylaxis.

The mandibular right second premolar was noted clinically in the position normally occupied by the first molar (Figure 2A); no first molar was present clinically or radiographically in its expected location, and the patient′s history did not clarify whether this reflected prior extraction, congenital absence, or failure of eruption. Multiple carious teeth and defective restorations were also present.

Periapical and panoramic radiographs (Figure 3) confirmed the second premolar occupying the first molar space and demonstrated generalized alveolar bone loss affecting both arches. No periapical pathology, root resorption, or other developmental anomaly was identified in relation to the displaced tooth. Based on the clinical and radiographic findings, a working diagnosis of transposition of the mandibular right second premolar and first molar was made, and a comprehensive treatment plan was formulated to address the patient’s chief complaint alongside the incidental findings.

**Figure 3:**
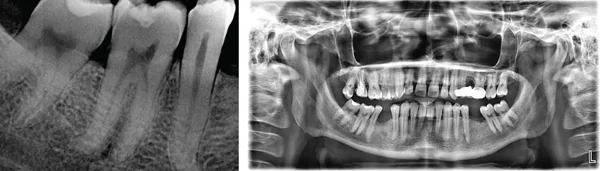
IOPA and OPG showing the mandibular right second premolar occupying the first molar space. The first molar was absent from its normal location; no interval or historical radiographs were available to establish the developmental history of this finding.

Management began with thorough scaling and oral prophylaxis (Figure 2), followed by restoration of carious teeth, and concluded with fabrication of prostheses for the edentulous spaces to restore masticatory function. The transposed premolar was retained in its current position without orthodontic or surgical intervention, as it was asymptomatic and posed no functional or esthetic concern.

## 3. Discussion

Tooth transposition is an uncommon anomaly, reported in approximately 0.3%–1.4% of the population, with most cases involving the maxillary canine and first premolar and a recognized female predilection [1, 4, 5]. In the present case, an adult male exhibited an anomalous position of the mandibular right second premolar within the first molar space—a finding that, when involving posterior teeth, is disproportionately underrepresented in the literature: A meta‐analysis of 120 published cases of tooth transposition found that first–second molar involvement accounted for less than 1% of reports, and to our knowledge no report specifically describing a complete premolar–first molar transposition in an adult could be identified for direct comparison [1]. This scarcity limits our ability to benchmark the present findings against an established comparator group and instead situates this case as a descriptive addition to a very small body of literature on posterior mandibular transpositions.

A central diagnostic question, raised during peer review of this report, is whether the present findings represent true developmental transposition or migration of the premolar into a space left by earlier loss of the first molar. Distinguishing these possibilities ideally requires longitudinal radiographs spanning tooth development, or cross‐sectional imaging demonstrating a root‐crown relationship consistent with a genuinely transposed root axis, neither of which was available in this case. The patient could not reliably recall whether the first molar had been extracted, was congenitally absent, or had failed to erupt, and no historical records were available to confirm this. We therefore present this case with appropriate caution: the clinical and panoramic radiographic appearance—a fully erupted premolar occupying the molar position, with no radiographic trace of the first molar—is consistent with transposition but cannot definitively exclude postextraction migration as an alternative explanation. We regard this diagnostic uncertainty as a limitation of the case rather than a settled conclusion, and we highlight it explicitly so that readers may weigh the evidence themselves.

Irrespective of the precise developmental mechanism, three observations are clinically noteworthy. First, the anomaly was entirely asymptomatic and was identified only incidentally during a comprehensive examination prompted by an unrelated complaint of masticatory difficulty; this reinforces the value of routine, thorough intraoral and radiographic assessment even when the presenting complaint does not point to the posterior mandible. Second, the patient′s periodontal findings—generalized gingival recession and alveolar bone loss—appear attributable to long‐standing plaque accumulation and poor oral hygiene rather than to the tooth malposition itself; no pattern of bone loss or recession localized specifically to the displaced premolar was identified that would suggest a causal relationship, and we interpret these as coincidental rather than related findings. Third, the malpositioned tooth did not appear to produce occlusal disharmony in this patient, likely because the adjacent edentulous spaces reduced the opportunity for interference; this contrasts with reports of anterior transpositions, which more frequently disrupt esthetics and occlusion given their location within the smile zone [2, 3].

Radiographic imaging remains central to diagnosis. Panoramic radiography, supplemented by periapical views or, where available, cone‐beam computed tomography, allows visualization of the three‐dimensional relationship between a displaced tooth and adjacent structures and is generally preferred over panoramic imaging alone when the diagnosis of true transposition needs to be firmly established [7]. In the present case, only panoramic and periapical radiographs were available; the absence of cross‐sectional imaging is acknowledged as a diagnostic limitation as discussed above.

The clinical message from this case is that atypical tooth positions in the posterior mandible can go undetected for years in adults when asymptomatic, and that their presence should be documented and factored into overall treatment planning even when no intervention targeting the anomaly itself is indicated. For the treating clinician, recognizing such findings avoids misinterpreting a displaced tooth as an isolated ectopic premolar and informs prosthetic planning for the edentulous space it now occupies.

In children, interceptive evaluation around 6–8 years of age with panoramic radiography can identify developing transpositions before they become fixed, potentially allowing repositioning that is no longer feasible once the permanent dentition and periodontium are fully established [8, 9]. This patient′s presentation in adulthood meant that interceptive treatment was not a consideration; management was therefore appropriately directed at the functional complaint through periodontal therapy, restorative care, and prosthetic rehabilitation, with the displaced premolar retained in its current position and monitored rather than surgically or orthodontically corrected, given the absence of any functional or esthetic indication for intervention.

## 4. Conclusion

This case describes a rare positional anomaly of the mandibular right second premolar within the first molar space, discovered incidentally in an adult patient. Although the clinical and radiographic findings are most consistent with tooth transposition, the absence of longitudinal imaging means postextraction migration cannot be entirely excluded, and this limitation is presented transparently. The case underscores the value of thorough clinical and radiographic examination in identifying atypical dental findings, even when asymptomatic, and highlights the importance of longitudinal or cross‐sectional imaging in confidently distinguishing developmental transposition from other causes of anomalous tooth position.

## Author Contributions

Sura Ali Ahmed Fuoad Albayati: conceptualization, supervision (clinical verification of diagnosis and management), writing—original draft, and writing—review and editing. Mohammed Issam Al Hilwani: investigation (clinical examination and treatment of the patient, data collection), visualization (preparation of radiographic figures), project administration (coordination of the manuscript revision and response to reviewers), and writing—review and editing. Faris Mohammad Abdoh: investigation (clinical examination and treatment of the patient, data collection).

## Funding

No funding was received for this manuscript.

## Ethics Statement

Ethical approval was not required for this single anonymized case report. According to the regulations of the authors′ institution, ethics approval is not required for retrospective case reports involving a single patient when all patient identifiers have been removed and the study does not alter or interfere with routine clinical practice. Written informed consent was obtained from the patient for publication of this case report and any accompanying images; a copy of the signed consent form is retained by the corresponding author and is available upon request. This report was prepared in accordance with the Declaration of Helsinki.

## Conflicts of Interest

The authors declare no conflicts of interest.

## Data Availability

The data that support the findings of this study are available on request from the corresponding author. The data are not publicly available due to privacy or ethical restrictions.
